# Training of Nurses in Cottage Hospitals

**Published:** 1924-11

**Authors:** 


### TRAINING OF NURSES IN COTTAGE HOSPITALS.

The General Nursing Council has approved a scheme submitted by the Royal United Hospital, Bath, under which probationers in Cottage Hospitals in parts of Wilts and Somerset will be able to take up while there the preliminary examination of the Council, under which two years' work in a Cottage Hospital may count as one of the three required for registration. Such approved Cottage Hospitals as may enter the scheme will be affiliated with the Royal United Hospital, Bath. Probationers of these hospitals must during the first four months undergo at the Cottage Hospital the course of instruction set out in Sections 1, 2, 4 and 5 of the Syllabus for the first year of training. During the following twenty months they will have to attend lectures of the sister tutor at the Royal United Hospital, Bath. These lectures will be given at that Hospital or at certain Cottage Hospitals and at the most convenient times. At the end of this period of training and lectures a probationer will be qualified to enter for the preliminary examination, and the time thus occupied will, for the purposes of registration, be considered equivalent to one year in a completed training school. There must be an undertaking on behalf of the probationer that after passing her preliminary examination she will carry out the third and fourth years of her training at the Royal United Hospital, Bath, or at some other recognised training school for nurses. This undertaking will not apply in the case of those nurses who intend to be trained as midwives only.

### The Selected Hospitals.

In order to arrange details as to the places where, and the times at which, the lectures will be given, and the payment of expenses, it will be necessary to hold a meeting of the matrons or other representatives of the hospitals concerned. The Council has approved of the following hospitals:—Chippenham, Frome, Melksham, Paulton, Savernake, Shepton Mallett, Swindon Victoria, Trowbridge, Warminster.

